# Accelerated Resolution of Pacemaker Pocket Hematoma With Adjunctive Phototherapy: A Case Report

**DOI:** 10.7759/cureus.85378

**Published:** 2025-06-04

**Authors:** James R Kneller

**Affiliations:** 1 Cardiology and Cardiac Electrophysiology, University of Arizona College of Medicine-Phoenix, Phoenix, USA

**Keywords:** complementary alternative medicine, complementary and integrative health, energy medicine, human biofield, interventional pain medicine, photobiomodulation, phototherapy modalities, surgical wound complications, wound healing and tissue repair, wound healing enhancement

## Abstract

Pocket hematoma remains a common complication of pacemaker procedures, causing patient discomfort and functional limitation while predisposing to device infection. A cutaneous phototherapy device known as X39^®^ (LifeWave Inc., Draper, Utah, United States) activates endogenous stem cells through the elevation of glycyl-L-histidyl-L-lysine (GHK) and is found to increase healing rate by 50% following pacemaker extraction. The Carnosine^®^ device (LifeWave Inc., Draper, Utah, United States) activates carnosine dipeptides throughout the body, which act as a high-energy phosphate system that enhances muscle function. The Glutathione^®^ device (LifeWave Inc., Draper, Utah, United States) dramatically boosts levels of glutathione, which supports tissue development and renewal. These skin patches contain natural substances that reflect rays of infrared light from the body's biofield, each having unique biological activity. The present study explores whether the combination of X39^®^, Carnosine^®^, and Glutathione^®^ devices may further accelerate wound healing.

A case of pacemaker pocket hematoma complicating the treatment of pneumothorax following a challenging pacemaker implantation procedure was presented in this report. In addition to standard care, devices were placed around the wound and replaced daily. Complete hematoma resolution was achieved after six days, which was >500% faster than expert consensus estimates. In conclusion, amplification of stem cells, carnosine, and glutathione activity appeared to perform synergistically, as healing occurred sixfold faster with this adjunctive therapy. This drug-free technology may be used to accelerate healing following surgical procedures.

## Introduction

Pocket hematoma is a frequent and dreaded complication of pacemaker and implantable cardioverter-defibrillator (ICD) procedures. Hematoma is often a source of significant patient morbidity and predisposes to device infection [1]. Drainage introduces additional infection risk and is only advisable if pocket exploration is needed to address an active source of bleeding. Management otherwise consists of waiting for the coagulum to gradually resorb. 

The benefits of cutaneous phototherapy devices (LifeWave Inc., Draper, Utah, United States) for wound healing and enhanced myocardial function have recently been demonstrated [2-4]. For example, complications of transradial access (TRA) for coronary angiography were mitigated using the IceWave® system [2]. Application of the first set of patches provided near-instantaneous relief of pain, sensory deficits, and impaired motor function due to radial nerve injury. Wound healing following the removal of an infected pacemaker requiring extensive pocket debridement was also found to progress 50% faster with the daily use of the X39® device [3]. Finally, placement of Carnosine® patches over the left thorax was shown to improve left ventricular ejection fraction (LVEF) by an average of 6.3% (p<0.05; n=30 subjects) within 30 minutes [4].

## Case presentation

**Figure 1:**
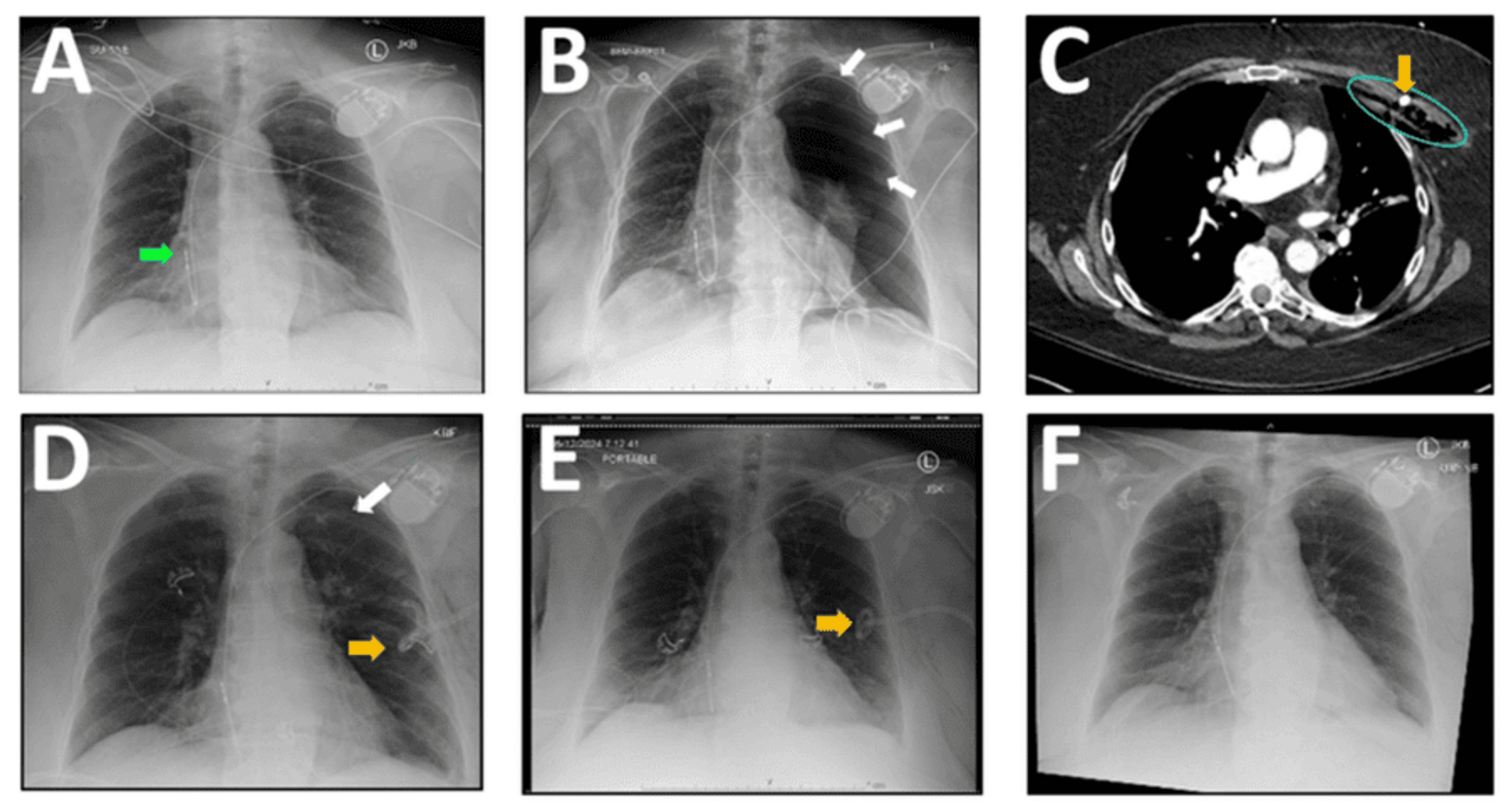
Evolution of pneumothorax Post-op chest X-ray showing the successful placement of a single-chamber atrial pacemaker, with the lead tip (green arrow) indicated (A). The patient presented with pneumothorax (white arrows) five days later (B). CT scan of the chest showed extensive subcutaneous air (blue circle) surrounding the pacemaker generator (C). Pneumothorax was treated with a pigtail catheter (orange arrow), with residual pneumothorax evident on post-op day 3 (D). Resolution of pneumothorax was evident on post-op day 5, indicating that the removal of the pigtail catheter (orange arrow) was appropriate (E). Chest X-ray on post-op day 6 confirmed stable resolution of pneumothorax (F), allowing for patient discharge from the hospital. CT: computed tomography

This report features a 76-year-old woman with morbid obesity (BMI 46 kg/m^2^), obstructive sleep apnea, hypertension, and paroxysmal atrial fibrillation with sick sinus syndrome referred for pacemaker therapy. Given her challenging body habitus, a 6-inch Tuohy needle was required for venous access. Post-op chest X-ray showed satisfactory device placement with no discernible complications (Figure 1 (A)). She presented five days later with sudden dyspnea, and chest X-ray revealed an ipsilateral pneumothorax (Figure 1 (B)), which was further delineated by computed tomography (CT) scan (Figure 1 (C)), demonstrating large pockets of subcutaneous air adjacent to the site of vascular access. A pigtail catheter was inserted into the ipsilateral chest cavity on an emergent basis, allowing for the evacuation of the trapped air (Figure 1 (D)).

**Figure 2:**
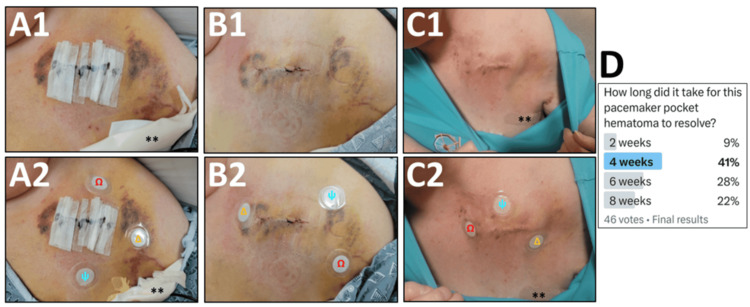
Acceleration of hematoma resolution with phototherapy devices Hematoma at baseline (A1), with improvement on day 3 (B1) and complete resolution after six days (C1). Phototherapy patches were placed along the perimeter of the hematoma (A2-C2), including X39^®^ (ѱ), Glutathione^®^ (Ω), and Carnosine^®^ (Δ). New patches were applied daily and rotated around the wound. Survey results for estimated healing time (D), obtained using the social media platform "X" (previously Twitter).  Hematoma resolution was sixfold faster than anticipated by expert consensus. External patch diameter is 34 mm. **: dressing for pigtail catheter (see text for details)

A large pacemaker pocket hematoma was also apparent (Figure 2 (A1)). Ultrasound of the pacemaker pocket revealed an elongated hypoechoic thrombus measuring 8 cm along the chest wall, 5 cm in width and up to 3 cm deep, although borders were obscured by the surrounding adipose tissue. Conservative management was deemed most appropriate. Given the patient's preference for complementary and alternative medicine (CAM), three phototherapy devices were utilized simultaneously to address the hematoma (Figure 2 (A2-C2)). These included the X39®, Carnosine®, and Glutathione® patches, which were applied daily on a rotational basis along the perimeter of the hematoma.

The patient was assessed for side effects, and patch stability was visually confirmed on a daily basis. Good progress was noted on day 3 (Figure 2 (B1-B2)), with complete resolution of the hematoma achieved within six days (Figure 2 (C1-C2)). There were no adverse or unanticipated events. Resolution of the pneumothorax was also surprisingly fast (Figure 1 (E)), permitting the early removal of the pigtail catheter on day 5 (Figure 1 (F)). Ancillary benefits from phototherapy included relief from arthritis pain, improved balance and appetite, and more restorative sleep, as previously described with X39® [5].

Only routine outpatient pacemaker management was subsequently required. The patient was happy that her pain and dyspnea resolved so quickly and that her hospital stay was shorter than anticipated. She appreciated the cosmetic difference (Figure 2 (A1) vs. Figure 2 (C1)) and was relieved that no infection occurred.

Rates of wound healing are not standardized, and comparison between cases is complex [3]. A survey was therefore conducted using the social media platform "X" (previously Twitter), inviting the electrophysiology community to estimate the time elapsed between Figure 2 (A1) and Figure 2 (C1). Blinded to the use of intervention, the weighted average estimate across respondents (n=46) was 5.3 weeks. Given an actual difference of six days, the time to resolution was sixfold faster than expert predictions (Figure 2 (D)).

## Discussion

The field of cardiac optogenetics utilizes focused pulses of light to activate proteins having optical sensitivity within the heart muscle, influencing myocardial physiology with unparalleled resolution. For example, low-intensity light may resolve aberrant heart rhythms [6]. This technology exploits the biological influence of photons, but is constrained by the need for external light sources. In contrast, phototherapy devices from LifeWave Inc. use the light energy that arises spontaneously from the body to influence physiologic processes. These adherent phototherapy devices measure 34 mm in diameter.

The mechanism of action is non-transdermal, meaning no substances are absorbed by the skin. The patches contain a reflective crystal made up of organic substances, salt, sugar, stabilized oxygen, and plant-based amino acids [5]. The human body continuously emits radiant energy in the near-infrared spectral range [7]. These emitted photons arise from the energetic biochemical reactions taking place simultaneously in the 50 trillion cells of the human body. The reflected light is most effective when the patches are placed over certain acupuncture points, where light is best transmitted to elicit physiologic effects [5]. The cost of these phototherapy devices was approximately $40.00.

The X39® device elevates levels of glycyl-L-histidyl-L-lysine (GHK) [8]. This peptide is known to activate dormant stem cells and stimulate dermal fibroblasts, thereby stimulating angiogenesis and neurogenesis while increasing the synthesis of collagen and glycosaminoglycans. Carnosine® patches increase the activity of endogenous carnosine throughout the body, which acts as a high-energy phosphate system supporting glycolysis and mitochondrial function [9]. Glutathione® patches from LifeWave Inc. stimulate production by the liver, raising systemic levels of glutathione by 300% [10]. This master antioxidant is vital for hundreds of physiologic processes, including tissue building and repair. As anticipated, this combination of phototherapy devices was found to synergistically contribute to rapid wound healing.

## Conclusions

Using a wearable phototherapy technology, a sixfold faster time to resolution for a large pacemaker pocket hematoma was observed, as determined by expert consensus survey data. Resolution of an associated pneumothorax was also more rapid than expected, shortening the duration of chest tube placement. Ancillary benefits of phototherapy included relief from arthritis pain, improved balance and appetite, and more restorative sleep. This case supports the synergistic benefit of activating multiple phototherapeutic pathways at once, achieved with the simultaneous placement of several different kinds of patches around a wound.

As a single case, the present findings are hypothesis-generating rather than mechanistic in nature. Causality cannot be inferred. Wellness products from LifeWave Inc., including the phototherapy patches used here, have not been evaluated by the US Food and Drug Administration and are not intended to treat medical conditions.
